# Large-Scale Conformational Changes of *Trypanosoma cruzi* Proline Racemase Predicted by Accelerated Molecular Dynamics Simulation

**DOI:** 10.1371/journal.pcbi.1002178

**Published:** 2011-10-13

**Authors:** César Augusto F. de Oliveira, Barry J. Grant, Michelle Zhou, J. Andrew McCammon

**Affiliations:** 1 Departments of Chemistry and Biochemistry, Howard Hughes Medical Institute, Center for Theoretical Biological Physics, University of California, San Diego, La Jolla, California, United States of America; 2 Department of Pharmacology, University of California, San Diego, La Jolla, California, United States of America; Stanford University, United States of America

## Abstract

Chagas' disease, caused by the protozoan parasite *Trypanosoma cruzi* (*T. cruzi*), is a life-threatening illness affecting 11–18 million people. Currently available treatments are limited, with unacceptable efficacy and safety profiles. Recent studies have revealed an essential *T. cruzi* proline racemase enzyme (TcPR) as an attractive candidate for improved chemotherapeutic intervention. Conformational changes associated with substrate binding to TcPR are believed to expose critical residues that elicit a host mitogenic B-cell response, a process contributing to parasite persistence and immune system evasion. Characterization of the conformational states of TcPR requires access to long-time-scale motions that are currently inaccessible by standard molecular dynamics simulations. Here we describe advanced accelerated molecular dynamics that extend the effective simulation time and capture large-scale motions of functional relevance. Conservation and fragment mapping analyses identified potential conformational epitopes located in the vicinity of newly identified transient binding pockets. The newly identified open TcPR conformations revealed by this study along with knowledge of the closed to open interconversion mechanism advances our understanding of TcPR function. The results and the strategy adopted in this work constitute an important step toward the rationalization of the molecular basis behind the mitogenic B-cell response of TcPR and provide new insights for future structure-based drug discovery.

## Author Summary

There is an urgent need for the development of better drug therapies for tropical diseases, including Chagas' disease, sleeping sickness and leishmaniasis. Known collectively as the human trypanosomiases, these traditionally neglected diseases are responsible for substantial human suffering and death in Latin America and sub-Saharan Africa. Current chemotherapy for Chagas' disease is particularly unsatisfactory, with available drug treatments displaying poor efficacy and undesirable toxic side effects. Recent developments in the study of the basic biochemistry of the causative *Trypanosoma cruzi* parasite and its host infection mechanism have identified an essential proline racemase enzyme (TcPR) as a novel target for chemotherapeutic intervention for Chagas' disease. Conformational changes associated with substrate binding to TcPR are believed to expose critical residues that elicit a host mitogenic B-cell response, a process contributing to parasite persistence and the undermining of host immunity against *T. cruzi*. Here we describe advanced accelerated molecular dynamics simulations that capture previously uncharacterized large-scale motions of TcPR. These motions reveal new conformational epitopes of potential importance for the mitogenic B-cell response. Furthermore, knowledge of the conformational interconversion mechanism and corresponding transient binding pockets will greatly aid future structure-based drug discovery efforts.

## Introduction

The protozoan diseases African sleeping sickness, leishmaniasis and Chagas' disease are responsible for substantial human suffering and death. Caused by parasites from the genus *Trypanosoma* these insect spread diseases mainly affect the underprivileged in tropical regions [1]. Limited drug therapies, human migration and environmental changes have contributed to the increasing spread of these traditionally neglected diseases. Chagas' disease, caused by the *Trypanosoma cruzi* parasite (*T. cruzi*), threatens the lives of millions of people from southern USA to southern Argentina [1], [2]. The need for new drugs is urgent with current treatments having poor efficacy and safety profiles, particularly in the late stage of the disease when the parasite has infected critical organs.

Recent studies have revealed an essential *T. cruzi* proline racemase enzyme (TcPR) as an attractive new candidate for chemotherapeutic intervention [3]. TcPR catalyzes the reversible stereoinversion of L- and D-proline [4]. Tonelli *et al.* showed that L-proline is essential for the intracellular differentiation of *T. cruzi*. [5]. Later, Chamond *et al.* demonstrated that over-expression of TcPR increases parasite differentiation into infective forms and its subsequent penetration into host cells [6]. In another study, Coatnoan *et al.* observed that, in addition to free D-amino acids, parasite extracts contain peptides composed of D-proline; indicating a possible mechanism used by the parasite to confer resistance against host proteolytic mechanisms [7].

TcPR has also been characterized as a potent host B-cell mitogen that sustains parasite evasion of specific host immune responses [3], [8]. B-cell proliferation and polyclonal antibody activation constitute a widespread mechanism of immune evasion shared by many pathogens. This process compromises immune response activation through generation of non-pathogen-specific B-cells that effectively mask specific reactions against the invading pathogen. In Chagas' disease, B-cell proliferation has also been linked with resistance to infection, disease progression and the pathology associated with its chronic form [3]. Taken together, these data provide strong evidence that TcPR represents a promising target for therapies that may more efficiently combat Chagas' disease.

**Figure 1:**
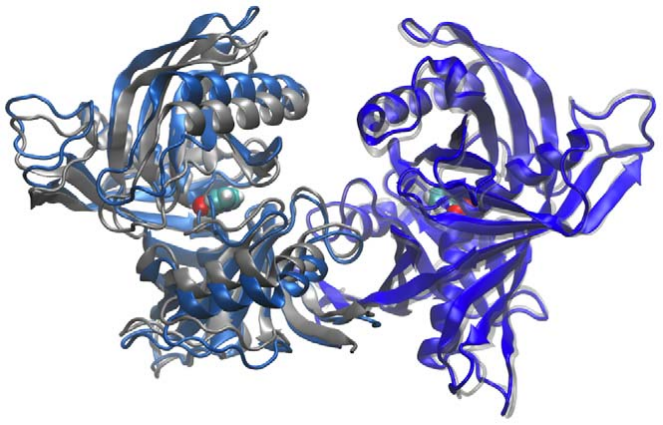
Available structures of TcPR indicate the occurrence of a closure movement induced by inhibitor binding. The homodimeric structures (PDB codes: 1W61 and 1W62) are superposed on monomers with a common closed conformation (dark blue and light gray chains, right of figure). Note the presence of a bound PYC inhibitor (spheres). A semi-open conformation is apparent in the absence of bound ligand (light blue monomer, left of figure).

Emerging crystallographic and mutagenesis data indicate that ligand-induced conformational changes in TcPR modulate the exposure of critical residues that elicit a host mitogenic B-cell response [3], [9]. Two crystal structures of TcPR are currently available (Figure 1). Each structure was solved with the transition state analog pyrrole-2-carboxylic acid (PYC) bound to either one or both monomers. In the presence of PYC monomers display a common closed conformation. In contrast a semi-open conformation is apparent for the ligand-free monomer (Figure 1). Together with calorimetric studies, these results indicate that a large inter- and intra- domain closure movement is coincident with ligand binding [9]. Although enzymatic inhibition by PYC abolishes mitogenic activity, point mutations of the catalytic cysteine residues (C130S and C300S) have little or no effect. Therefore, these studies not only showed that the mitogenic and enzymatic activity of TcPR are decoupled, but also strongly indicate that ligand-induced conformational changes upon binding prevent the interaction of TcPR with B-cell receptors.

Here we show for the first time a model for the open form of TcPR. Characterization of the opening transition required the application of state of art accelerated molecular dynamics simulations, which extends the effective simulation time scale of conventional molecular dynamics. When combined with sequence conservation and small molecule fragment mapping analyses our results indicate that the mitogenic properties of TcPR are likely associated with the exposure of conserved conformational epitopes located around previously unidentified binding pockets. This work represents an important step toward the rationalization of the molecular basis of TcPR initiated B-cell response and provides a basis for future structure-based drug discovery.

## Results/Discussion

Characterization of the opening movement of TcPR requires access to long-time scale, inter-domain motions that are currently inaccessible by conventional molecular dynamics (MD) simulations [10]. To overcome this limitation, we applied an enhanced sampling technique developed in our group, called accelerated molecular dynamics (aMD), which extends the effective simulation time scale. In aMD, a continuous non-negative boost potential function, ΔV(r), is added the original potential surface (V(r)) such that regions around the energy minima are raised and those near the barriers or saddle points are left unaffected. ΔV(r) is defined according to ΔV(r) = (E−V(r))^2^/(α+E−V(r)). Whenever V(r) is below a chosen threshold boost energy, E, the simulation is performed on the modified potential V*(r) = V(r)+ΔV(r), otherwise sampling is performed on the original potential V*(r) = V(r). The parameter α modulates roughness and the depth of the energy minima on the modified surface, as previously shown (see materials and methods for details) [11]–[17].

**Figure 2:**
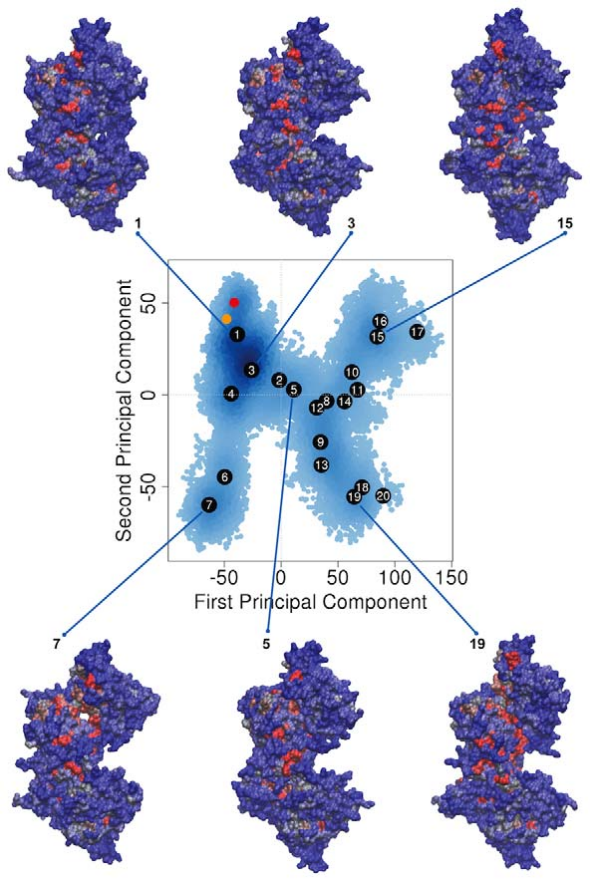
Principal component analysis characterizes large-scale inter- and intra-domain opening motions of apo TcPR. Projection of instantaneous aMD trajectory conformers on their first and second principal components. The distribution of MD conformers is depicted with density-shaded blue points (dark: high density, light: low density). The available crystal structures (1W61: red and 1W62: orange) along with central members of dominant conformational clusters are numbered (from **1** to **20**) along with selected molecular surface representations colored by sequence conservation (red: high, blue: low, see also Video S1).

The closed crystal structure of TcPR in complex with two transition-state analog inhibitors (PDB code: 1W61) was used to build our initial model. Atomic coordinates of bound PYC were removed from the active site of each monomer resulting in a ligand-free closed system that underwent 100 ns of aMD simulation. To characterize dominant conformational states, along with inter- and intra-domain opening motions, the final aMD trajectory was subjected to principal component analysis (PCA) [14], [18]. Figure 2 displays the two-dimensional representation of the structural dataset as a projection of the distribution onto the subspace defined by the first and second principal components. Large-scale opening motions of TcPR were well characterized and captured by PC1 and PC2 (which together with PC3 accounted for over 70% of the variance in the original distribution: see Figure S1). Clustering of trajectory conformers was used to visualize the dominant conformations sampled by the simulation (Figures 2 and Figure S2). Two major clusters, encompassing the closed and open conformational states, are clearly identified in the ensemble of conformers. Six representative structures, which include closed and open cluster representatives of TcPR, are displayed in Figure 2. The TcPR structures are shown in molecular surface representation colored according to the level of residue conservation within the proline racemase family (with blue and red representing low and highly conserved residues respectively, see materials and methods for details).

**Figure 3:**
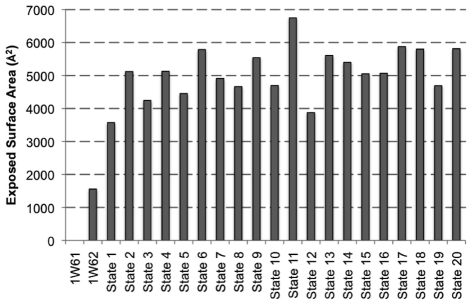
Exposed Surface Area. Exposed surface calculated as the difference between the solvent accessible surface of trajectory representative states (**1** to **20**), and the closed form of TcPR (1W61).

Projection of the two available crystal structures onto the PCs obtained from the aMD trajectory reveals that both closed and semi-open forms of TcPR are well characterized by the conformers sampled in the vicinity of state **1** (Figure 2), indicating that significantly larger opening motions are observed in the trajectory. Projection of TcPR aMD trajectory onto PC sub-space characterized two dominant global motions: (a) A large-scale inter-domain motion that exposes several conserved residues located in the monomer-monomer interface, and that is observed when the conformer population shifts from state **1** to states **15**, **16** or **17** (moving along PC1) and (b) a large-scale intra-domain opening movement that exposes highly conserved segments around the active site region of TcPR, and that is observed when the system shifts population from state **1** to **7** (moving along PC2). Combination of these two global motions leads TcPR to regions around states **18**, **19** and **20**. These states represent some of the most open structures accessed by aMD and display the newly identified and highly solvent exposed regions at both monomer-monomer interface and in the surrounds of the active site (see Video S1). As shown in Figure 3, the surface area exposed by states **1** to **20** is dramatically larger than the one presented by the semi-open crystallographic structure 1W62. For instance, the large conformational change involved in the formation of the bound complex from open states **6**, **17**, **18** or **20** buries an area of ∼6000 Å^2^; corresponding to approximately four times the buried area observed in crystal structures 1W61 and 1W62 (∼1500 Å^2^) [9]. To better visualize the magnitude of the long-time large-scale conformational changes, states **1** (closed) and **19** (open) were compared to the superimposed crystal structures (See Videos S2 and S3). It is worth noting that the amplitude of the motion associated with the experimentally observed conformational change is significantly smaller than the ones associated with the inter- and intra-domain motions obtained in our aMD simulation.

To further understand the physical basis of the observed opening motions, we analyzed the available structures with a simplified elastic-network normal mode method [19]. In the elastic network approach, a single model (expressed in terms of Cα coordinates) leads to an objective expression of possible protein dynamics in terms of a superposition of collective normal mode coordinates [20]. The structural mobility predicted by Normal Modes Analysis (NMA) performed on the semi-open structure (PDB ID 1W62) revealed a high overlap between the lowest three modes and the eigenvectors obtained from aMD simulations (0.6 for mode 1 to PC1, see Figure S3). This result indicates that the dominant collective motions during aMD simulation, that capture the TcPR opening movement, are indeed low-frequency motions intrinsic to the structure.

**Figure 4:**
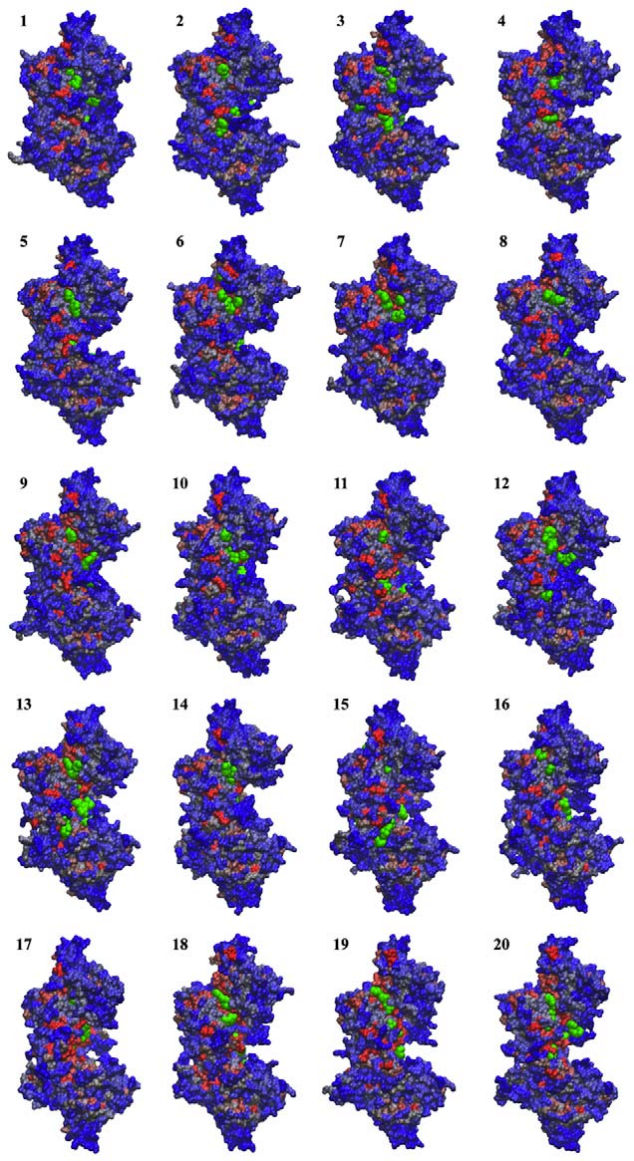
Potential B-cell interaction sites identified through fragment mapping analysis (probe molecules shown in green). Representative conformers from cluster **1** to **20** are shown in molecular surface representation and colored by sequence conservation (red: high, blue: low).

**Figure 5:**
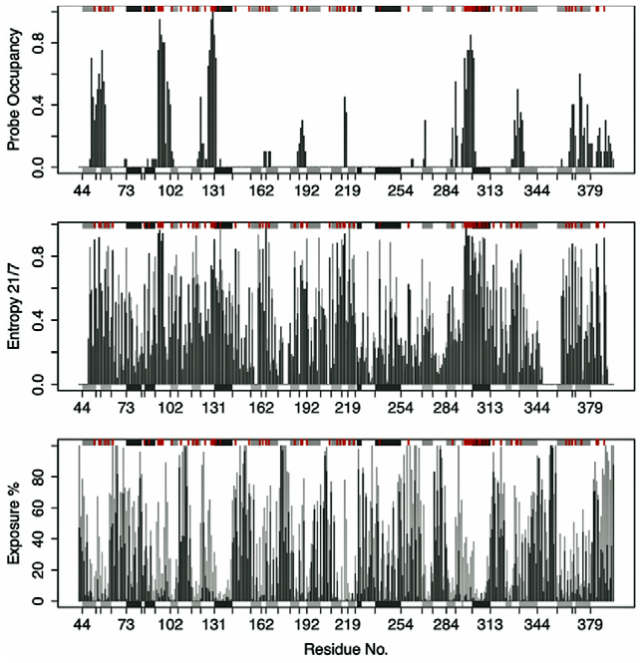
Probe binding occupancy, sequence conservation and solvent exposure per reside in TcPR. (a) Potential probe binding site, or “hot-spot”, residues across trajectory conformers (b) Sequence entropy scores for both a 21-letter alphabet (20 amino acids and a gap) and 7- letter alphabet (where amino acids are grouped into 6 classes based on their physicochemical properties) are plotted per position in dark gray and light gray, respectively. (c) The mean and maximum solvent exposure per position in all trajectory structures (in dark and light gray respectively). The major elements of secondary structure (shaded rectangles) and positions with a high degree of sequence conservation (red ticks) are indicated in the marginal areas of each plot to facilitate comparison.

As previously noted, in vitro assays of B-cell proliferation together with structural information strongly indicate that the closure movement induced by ligand binding prevents the interaction of TcPR with B-cell receptor molecules [3], [9]. Activation of B-cell polyclonal response is likely to be associated with the occurrence of transient binding pockets, along with conformational epitopes, in the open ligand-free form of TcPR. In order to identify potential B cell binding sites in the newly identified open states, we used a fragment-based approach (FTMAP) to map binding hot spots on each of the twenty dominant trajectory conformers [21]. Based on the ideas behind screening small organic fragments by NMR and X-ray crystallography, FTMAP correlates pocket druggability with their propensity to bind clusters of small organic compounds. Figure 4 displays the mapping results for states **1** to **20**. To further characterize the location of each hot spot, probe occupancy was calculated and assigned to each residue of TcPR (see materials and methods for details). Figure 5a displays the final probe occupancy values obtained after combining and normalizing the results from all twenty conformational states.

As expected, high probe occupancy values were obtained for sites around the catalytic cysteines (residues 130 and 300), consistent with the existence of this binding site in all states, **1** to **20**. Several additional pockets, displaying low and high occupancies, were also identified. It is worth mentioning that the large variation in probe occupancies reveals the intrinsic dynamic nature of these binding pockets. Nevertheless, residues showing low probe occupancy values identify regions on the protein surface where potential interaction sites are exposed only in the open form of TcPR, (this includes interaction sites in the vicinity of residues 186–191, 217–218 and 288–291). In order to quantify the exposed surface area of each residue associated with the opening movement, the percentage exposure was calculated based on the per residue solvent accessible surface area of each state and the closed form of the TcPR (1W61). As can be seen in Figure 3c, the newly identified binding pockets are indeed sites that become considerably more exposed in the open states (Figure 3c, light grey). Moreover, sequence conservation analysis shows that these binding pockets are also highly conserved in all proline racemases (Figure 3b).

X-ray crystallography and mutagenesis studies indicate that interaction with B-cell receptors is likely to be associated with the presence of transient binding pockets that are fully formed only in the ligand-free open TcPR. In this work, we show for the first time a model for the open form of this important drug target obtained through the application of state of the art molecular dynamic simulation. Additionally, our results indicate that the mitogenic properties of TcPR may be associated with the exposure of conformational epitopes located around the newly identified binding pockets. Experimental mutagenesis studies of these sites is required to verify their potential role in eliciting host B-cell responses. In summary, the strategy adopted in this work allowed the characterization of large-scale conformational changes associated with the dynamic formation of potential interaction sites coupled with the exposure of highly conserved regions of the protein surface (Figure 5). Furthermore, the results presented in this work represent the first attempt to rationalize the molecular basis of the mitogenic B-cell response to TcPR and provide a basis for future structure-based drug discovery.

## Materials and Methods

All simulations were performed with the AMBER10 package [22] and corresponding all- atom potential function ff99SB [23]. Unless otherwise noted, all additional analyses were performed with the Bio3D package (available from http://mccammon.ucsd.edu/~bgrant/bio3d/) [18].

### Molecular dynamics

The crystal structure of Trypanosoma cruzi proline racemase (TcPR) in complex with 2 molecules of pyrrole-carboxilic acid (PDB code 1W61) was used to build our model. Initial atomic coordinates build of the apo form of TcPR was obtained by removing both inhibitors molecules from 1W61. In our model, basic residues like Arg and Lys are protonated, and acidic residues like Asp and Glu are deprotonated. Due to its normal pKa, the His residues were assumed to be neutral at physiological pH.

Initial energy minimization was performed by applying 500 steps of steepest descent followed by 500 steps of conjugate gradient minimization. The relaxed structures were then solvated in a truncated cubic box of pre-equilibrated TIP3P water molecules, which extended 10 Å further than the protein atoms. To neutralize the systems, sodium counterions (Na+) were added to balance the charge of the protein. The system was then energy minimized for 500 steps of steepest descent followed by 500 steps of conjugate gradient minimization using constant volume periodic boundaries. We kept the protein atoms and the ions fixed throughout the whole preparation process. In order to relax the protein in the solvent environment, all coordinates were optimized by employing 1000 steps of steepest descent followed by 1500 steps of conjugate gradient. After that, a 1 ns molecular dynamics (MD) simulation was preformed to heat the system from 0 K to 300 K, for which we applied the NVT ensemble (T = 298 K). To bring the systems to the correct density, we carried out a 100 ps MD simulation on which NPT ensemble (T = 298 K, P = 1 bar) was applied. For the production runs, we performed an additional Accelerated MD simulation (aMD) of 100 ns. The equations of motion were integrated every 2.0 fs using the Verlet Leapfrog algorithm. For analysis, the trajectory was sampled every 1.0 ps. During the MD runs, temperature and pressure were controlled via a weak coupling to external bath with a coupling constants of 0.5 and 1.0 ps, respectively. The center-of-mass motion was removed at regular intervals of 500 fs. The PME summation method was used to treat the long-range electrostatic interactions in the minimization and simulation steps of the solvated systems. The short-range nonbonded interactions were truncated using an 8 Å cutoff and the nonbonded pair list was updated every 20 steps. All calculations, conventional and accelerated MD simulations, were performed using an in-house modified version of AMBER10 package.

### Accelerated molecular dynamics

Accelerated MD approach modifies the energy landscape by adding a boost potential, Δ*V*(*r*), to the original potential surface every time *V*(*r*) is below a pre-defined energy level *E* [16], as
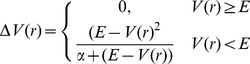
(1)where *α* modulates the depth and the local roughness of the energy basins in the modified potential. In principle, this approach also allows the correct canonical averages of an observable, calculated from configurations sampled on the modified potential energy surface, to be fully recovered from the accelerated MD simulations. In order to simultaneously enhance the sampling of internal and diffusive degrees of freedom a dual boosting approach was employed, based on separate torsional and total boost potentials as [15]

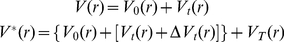
(2)where *V_t_*(*r*) is the total potential of the torsional terms, Δ*V_t_*(*r*) and Δ*V_T_*(*r*) are the boost potentials applied to the torsional terms *V_t_*(*r*) and the total potential energy *V_T_*(*r*), respectively. The parameters were set as follows. *E_t_* = 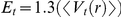, i.e. 30% higher than the ensemble-averaged torsional potential energy from conventional MD simulation. *α_t_*≈500 kcal mol^−1^ chosen based on previous work by de Oliveira [11]. *E_T_* = 0.2 kcal mol^−1^ (nr. atoms)^−1^ plus the ensemble-averaged total potential energy from conventional MD simulation. *α_T_*≈0.2 kcal mol^−1^ (nr. atoms)^−1^ [11]. These *E_T_* and *α_T_* values allow to reproduce the most relevant structural and energetic properties of liquid water while increasing the water self-diffusion coefficient by ∼15% [11], [15].

### Principal component analysis

Prior to trajectory superposition and Principal component analysis (PCA), iterated rounds of structural superposition were used to identify the most structurally invariant region. This procedure entailed excluding those residues with the largest positional differences (measured as an ellipsoid of variance determined from the Cartesian coordinates for equivalent Cα atoms of each frame), before each round of superposition, until only the invariant “core” residues remained [18]. This structurally invariant core consists predominantly of residues within secondary structure elements and was used as the reference frame for superposition of both crystal structures and subsequent MD trajectory snapshots. PCA was then employed to further examine inter-conformer relationships. The application of PCA to MD trajectories, along with its ability to provide considerable insight into the nature of conformational differences in a range of protein families has been previously discussed [14]. Briefly, PCA is based on the diagonalization of the covariance matrix, C, with elements Cij, built from the Cα atom Cartesian coordinates, r, of the superposed trajectory frames:
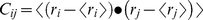
(3)where i and j represent all possible pairs of 3N Cartesian coordinates, where N is the number of atoms being considered. The highly mobile N and C-terminal residues (positions 42–43 and 380–398) were excluded from analysis as their high intrinsic mobility was found to mask the separation of the more pertinent open-to-closed domain displacements. The eigenvectors of the covariance matrix correspond to a linear basis set of the distribution of structures, referred to as principal components (PCs), whereas the eigenvalues provide the variance of the distribution along the corresponding eigenvectors. Projection of the distribution onto the subspace defined by the largest principal components results in a lower dimensional representation of the structural dataset. The percentage of the total mean-square displacement (or variance) of atom positional fluctuations captured in each dimension is characterized by their corresponding eigenvalue.

### Conformer clustering

**Figure 6:**
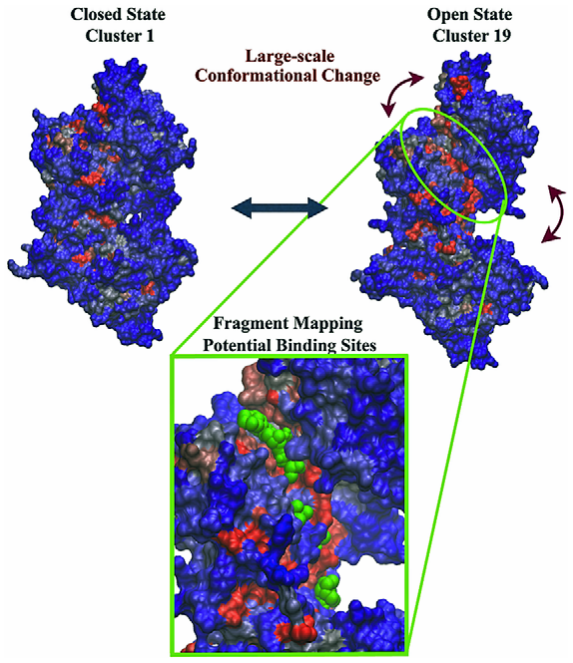
Large-scale opening of apo TcPR characterized by accelerated molecular dynamic simulation. Potential B-cell interaction sites were identified through fragment mapping analysis (probe molecules shown in green). Representative cluster **1** (closed state) and cluster **19** (open state) conformers are shown in molecular surface representation and colored by sequence conservation (red: high, blue: low).

Clustering of trajectory conformers was used to visualize the dominant conformations sampled by each simulation. Structures from aMD simulations underwent average-linkage hierarchical clustering according to the pairwise distances obtained from their projection onto the first 3 principal components. Clustering based on pairwise RMSD yielded similar major clusters. However, a significantly larger number of small clusters were returned due the influence of TcPRAC's highly flexible termini that do not contribute to the dominant principal components. Note that PCs 1–3 account for ∼70% of the variance in the original distribution (Figure 6) and produce a more succinct distance measure than the examination of average all-atom distances. This metric aids interpretation of an otherwise noisy signal as it is derived primarily from the concerted displacement of subdomains relative to one-another.

Inspection of the resulting dendogram was used to partition structures into 20 dominant groups (ranked according to their populations). The closest structure to the average structure from each cluster, in terms of RMSD, was chosen as a representative for further fragment mapping and virtual screening analysis described below.

### Sequence conservation analysis

To assess the level of sequence conservation at each position in the alignment, the similarity, identity, class identity and entropy per position were calculated. The “similarity” was defined as the average of the similarity scores of all pairwise residue comparisons for that position in the alignment (where the similarity score between any two residues is the score value between those residues in the BLOSSUM 62 substitution matrix [24]). The “identity” (i.e. the preference for a specific amino acid to be found at a certain position) was assessed by averaging the identity scores resulting from all possible pairwise comparisons at that position in the alignment (where all identical residue comparisons are given a score of 1 and all other comparisons are given a value of 0). The “class identity” was calculated in a similar manner to the “identity”. The exception being that amino acids were considered class identical (i.e. assigned a score of 1) if they possessed similar physicochemical properties. For this analysis residues were partitioned into three classes based on their relative hydrophobicity and the extent to which they are distributed between the surface and interior of known globular aqueously soluble protein structures (see [25], [26], and references therein). The first class contains hydrophobic residues (C, V, L, I, M, F and W) that have a high probability of residing within protein interiors. The second class contains hydrophilic residues (R, K, E, D, Q and N) that are most likely to be found on the surface of proteins. Finally, the third class is comprised of neutral residues (P, H, Y, G, A, S and T) that have a roughly equal chance of being on the surface or in the interior. “Entropy” is based on Shannon's information entropy for both a 21-letter alphabet (20 amino acids and a gap character) and a 7-letter alphabet (6 groups of amino acids and a gap character) [27], [28] (Equation 4):
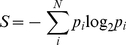
(4)where *S* is Shannon's entropy, *p_i_* is the frequency of each alphabet's letter at position *i* and *N* is the alphabet's size (7 or 21). The six groups chosen were aliphatic (A, V, L, I, M and C), aromatic (F, W, Y and H), polar (S, T, N and Q), positive (K and R), negative (D and E), and finally special conformations (G and P). Entropy scores plotted in Figure 3 are normalized so that conserved (low entropy) columns score 1 and diverse (high entropy) columns score 0 (Equation 5):
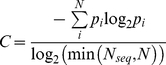
(5)where, *C* is the normalized entropy, *p_i_* is the frequency of each alphabet's letter at position *i*, *N* is the alphabet's size and *N_seq_* is the length of the sequence. We define a position to be conserved if the similarity, identity, class identity entropy 21 or entropy 7 at a position is >0.6. Positions in which more than 30% of the sequences had gaps were excluded from all sequence conservation analysis.

### Exposed Surface Area

Percent solvent exposure per position was calculated with the NACCESS program available at http://www.bioinf.manchester.ac.uk/naccess/. A residue was considered exposed when the accessible surface area (ASA) of the residue was more than 40% of the measured ASA of that residue in an extended G-X-G tripeptide context.

### Normal mode analysis

We employed the coarse-grained AD-ENM normal mode analysis approach developed by Zheng *et al.* [19]. AD-ENM implements a single-parameter Hookean potential, which has previously been shown to yield low-frequency normal modes that are in good agreement with those obtained from more detailed, empirical, force fields. For further details see [19], [20]

### Fragment mapping

We used the FTMap method of Brenke and co-works to highlight regions on the TcPR surface that have the potential to bind the highest number of small molecular probes [21]. Both crystal structures and each cluster representative form aMD were subject to fragment mapping. Hot-spot residues (those that comprise prominent fragment binding sites) were analyzed across all structures. A residue was assumed to be in contact with a probe molecule if any two heavy atoms from the probe and residue were closer than 5.0 Å.

## Supporting Information

Figure S1 **Results of PCA on the TcPR aMD trajectory.** (a–c) Conformer plots: Projection of trajectory structures onto the principal planes defined by the three most significant principal components (termed PC1–3). Structures are colored by time evolution (From blue to red). (d) Eigenvalue spectrum: Results obtained from diagonalization of the atomic displacement correlation matrix of Cα atom coordinates from the trajectory. The magnitude of each eigenvalue is expressed as the percentage of the total variance (mean-square fluctuation) captured by the corresponding eigenvector. Labels beside each point indicate the cumulative sum of the total variance accounted for in all preceding eigenvectors.
(TIF)

Figure S2 **Hierarchical clustering according to the pair-wise distances obtained from the projection onto the first three principal components.** The color bars showed in the x and y axes highlight sub-clusters of structures composing the two main clusters representing the open and closed conformational states.
(TIF)

Figure S3 **Normal mode analysis.** Overlap between the lowest three modes and the eigenvectors obtained from aMD simulations.
(TIF)

Video S1 Representative cluster conformers are shown in molecular surface and protein cartoon representations and colored by sequence conservation (red: high, blue: low, see text for details).
(MOV)

Video S2 Opening movement characterized by the superposition of the semi-open and closed crystal structures.
(MOV)

Video S3 Opening movement characterized by the superposition of states **1** (closed) and **19** (open).
(MOV)
