# Supplementary figures and images for: Large-Scale Conformational Changes of Trypanosoma cruzi Proline Racemase Predicted by Accelerated Molecular Dynamics Simulation
Source: PLoS Comput Biol. 2011 Oct 13;7(10):e1002178. doi: 10.1371/journal.pcbi.1002178 (PMC3192803; doi:10.1371/journal.pcbi.1002178)

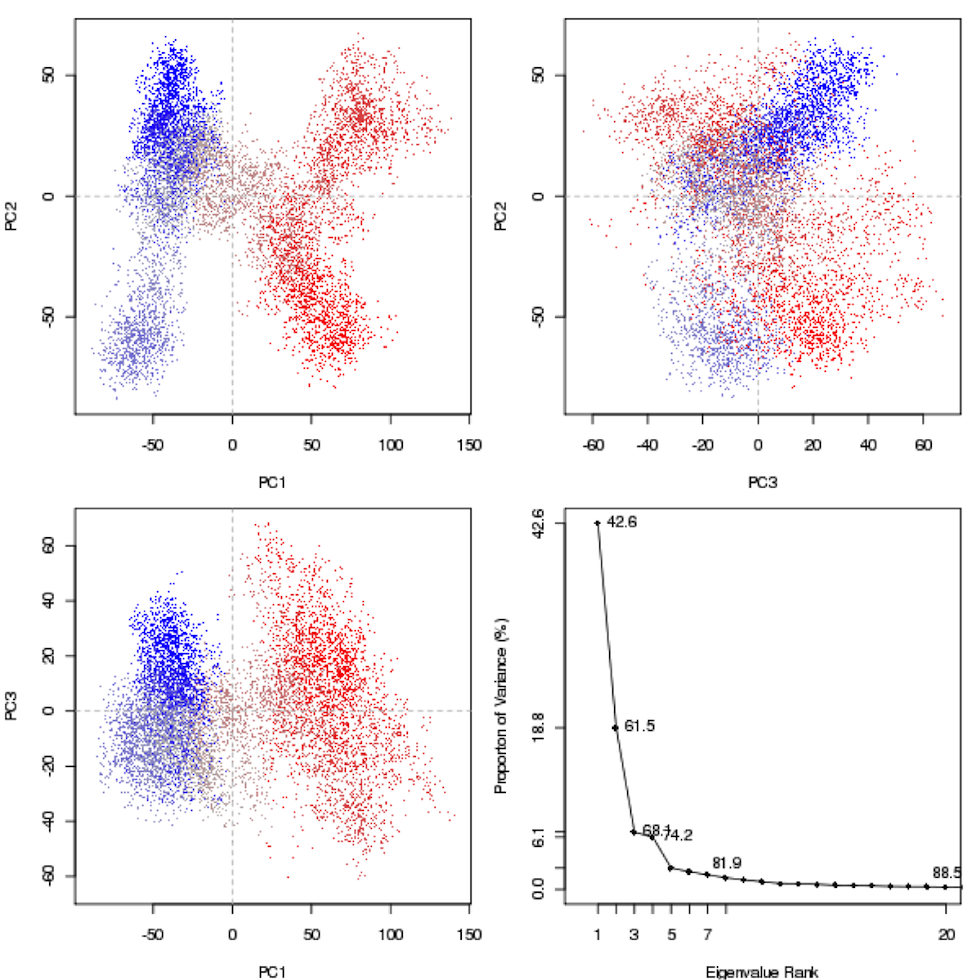

Supplement: Figure S1 — Results of PCA on the TcPR aMD trajectory. (a–c) Conformer plots: Projection of trajectory structures onto the principal planes defined by the three most significant principal components (termed PC1–3). Structures are colored by time evolution (From blue to red). (d) Eigenvalue spectrum: Results obtained from diagonalization of the atomic displacement correlation matrix of Cα atom coordinates from the trajectory. The magnitude of each eigenvalue is expressed as the percentage of the total variance (mean-square fluctuation) captured by the corresponding eigenvector. Labels beside each point indicate the cumulative sum of the total variance accounted for in all preceding eigenvectors. (TIF) [file pcbi.1002178.s001.tif]

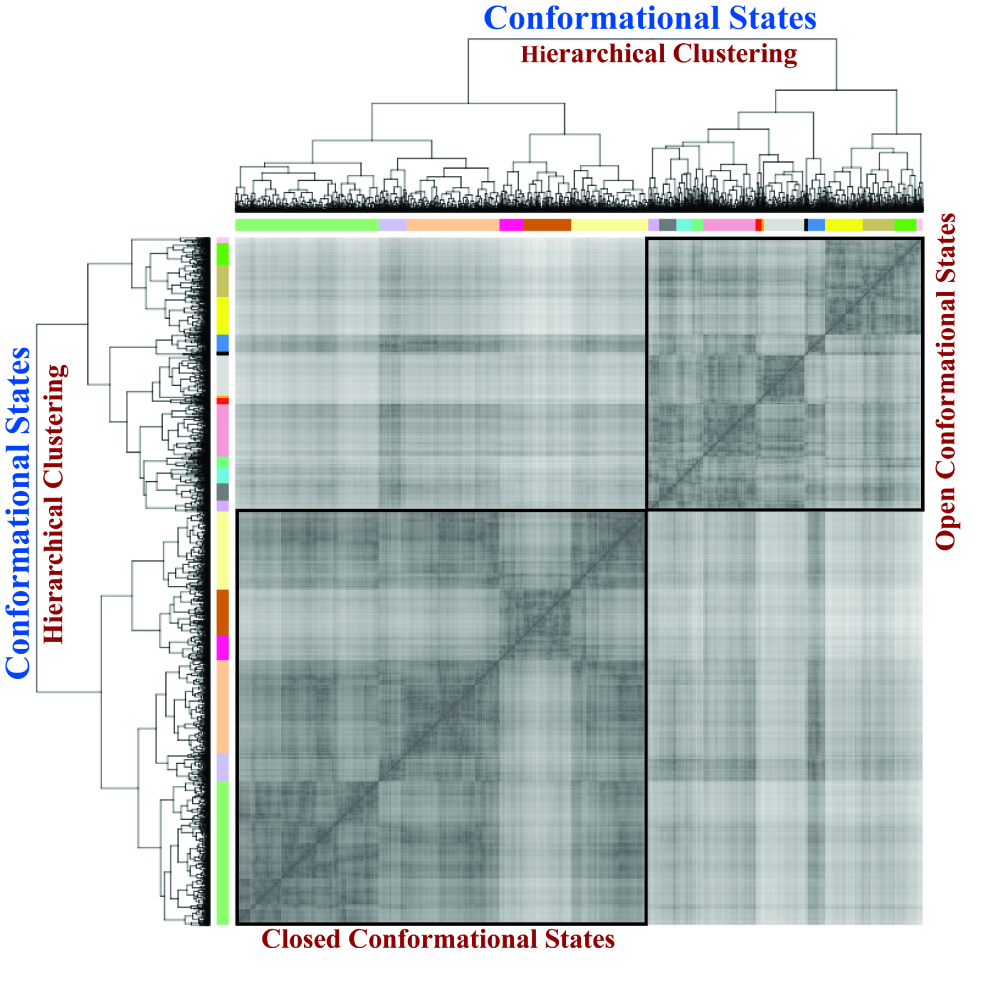

Supplement: Figure S2 — Hierarchical clustering according to the pair-wise distances obtained from the projection onto the first three principal components. The color bars showed in the x and y axes highlight sub-clusters of structures composing the two main clusters representing the open and closed conformational states. (TIF) [file pcbi.1002178.s002.tif]

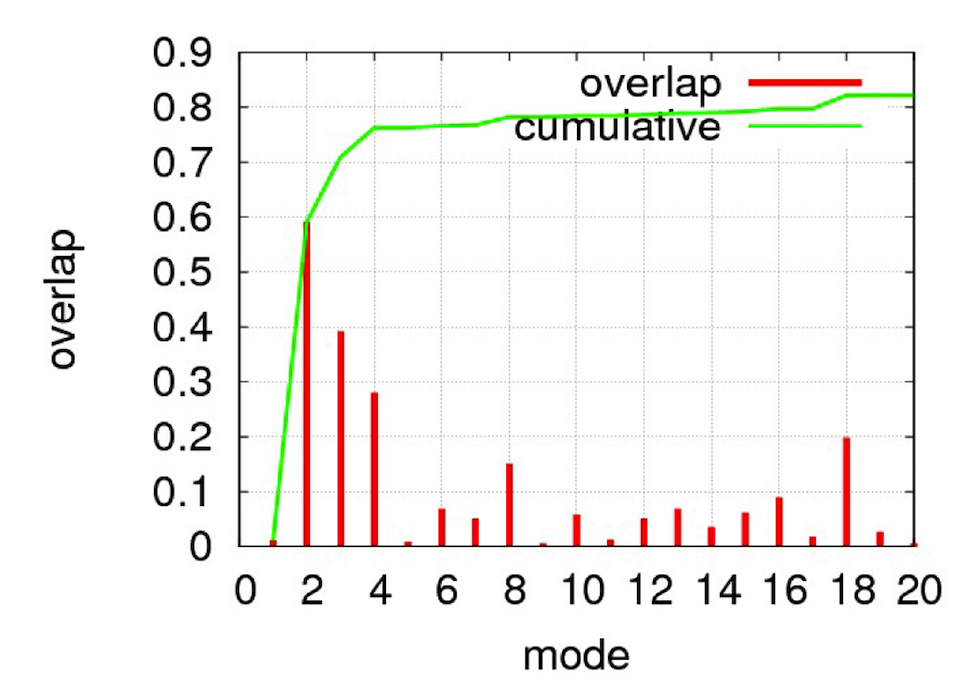

Supplement: Figure S3 — Normal mode analysis. Overlap between the lowest three modes and the eigenvectors obtained from aMD simulations. (TIF) [file pcbi.1002178.s003.tif]
